# Phos-fate of the liverwort: Unlocking the secrets to Pi homeostasis in *Marchantia polymorpha*

**DOI:** 10.1093/plphys/kiae522

**Published:** 2024-10-04

**Authors:** Jiawen Chen, Nicola Trozzi

**Affiliations:** Assistant Features Editor, Plant Physiology, American Society of Plant Biologists; Division of Crop Biotechnics, Department of Biosystems, KU Leuven, 3001 Leuven, Belgium; Assistant Features Editor, Plant Physiology, American Society of Plant Biologists; Department of Computational and Systems Biology, John Innes Centre, Norwich NR4 7UH, UK; Department of Plant Molecular Biology, University of Lausanne, CH-1015 Lausanne, Switzerland

Phosphorus (P) is a critical nutrient for plants, forming key molecules like ATP and phospholipids. Plants absorb P as inorganic phosphate (Pi), but Pi is often limited in soils, leading to widespread use of fertilizers in agriculture. Flowering plants, like Arabidopsis (*Arabidopsis thaliana*), rely on root systems to absorb and transport Pi through their vascular networks, allowing them to efficiently manage nutrient uptake from the soil. In contrast, nonvascular plants such as Marchantia (*Marchantia polymorpha*) lack both roots and vascular systems, raising the question of how they regulate Pi without these specialized structures.

To help manage Pi, plants use signaling molecules called inositol phosphates (InsPs), which are derived from the phosphorylation of inositol, a sugar. InsPs control Pi uptake, storage, and distribution. For instance, the fully phosphorylated InsP_6_ (phytic acid) is the primary P storage form in seeds. Inositol pyrophosphates (PP-InsPs) additionally have diphosphate groups attached to the inositol and are especially important for fine-tuning how plants adjust to Pi availability (Shears 2004). In flowering plants, the InsP kinase ITPK1 converts InsP_6_ to 5-InsP_7_, a crucial step in producing PP-InsPs, which help regulate phosphate homeostasis by controlling how the plant responds to low Pi levels (Laha et al. 2019; Riemer et al. 2021).

In this issue of *Plant Physiology*, Pullagurla et al. (2024) investigated whether nonvascular plants like Marchantia use similar mechanisms as Arabidopsis to manage Pi. They observed that Marchantia responds to low Pi availability by reducing thallus growth and increasing the number and length of rhizoids, which may parallel adaptations in flowering plants despite their structural differences (Rico-Reséndiz et al. 2020). The researchers generated Marchantia mutants that either overexpressed Mp*ITPK1* or lacked a functional Mp*PHO1a* gene, which is involved in Pi transport in Arabidopsis (Hamburger et al. 2002). In the Mp*ITPK1* overexpression lines, higher levels of 5-InsP_7_ and InsP_8_ were observed compared with wild-type plants, along with a weakened response to Pi starvation (Fig.), such as fewer and shorter rhizoids. However, no significant changes in thallus growth were observed between the wild type and Mp*ITPK1* overexpression lines under Pi starvation. The authors found that MpITPK1 could restore AtITPK1 Pi-related functions in Arabidopsis *atitpk1-2* lines, suggesting that Pi management strategies were established early in plant evolution, before the divergence of Marchantia and Arabidopsis.

**Figure.**
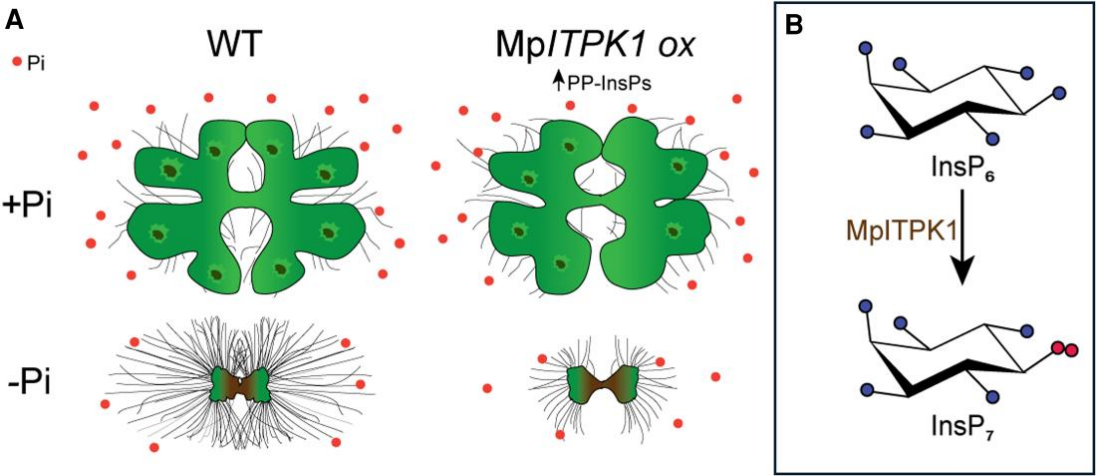
**A)** Comparison of wild-type (WT) and MpITPK1 overexpression (*MpITPK1 ox*) lines of Marchantia polymorpha under phosphate-sufficient (+Pi) and phosphate-deficient (−Pi) conditions. The drawings show the phenotypic impact on thallus area and rhizoid density in both conditions. **B)** The panel illustrates the chemical conversion of InsP_6_ to 5-InsP_7_, with MpITPK1 indicated as the catalyzing enzyme in the reaction. Adapted from figure by Naga Jyothi Pullagurla and Riya Ghosh.

The Mp*pho1a* knockout mutants also showed significant changes in Pi regulation. The Mp*pho1a* knockout lines accumulated more Pi in their rhizoids than in thallus tissue, suggesting MpPHO1a plays a role in directing Pi to different parts of the plant. These mutants also had abnormal levels of InsPs and PP-InsPs, particularly a large increase in 1,5-InsP_8_, indicating that both MpITPK1 and MpPHO1a are critical for maintaining Pi balance in Marchantia. The findings suggest that Pi homeostasis controlled by PP-InsPs is conserved across nonvascular and flowering plants.

The study also revealed that, similar to flowering plants, Marchantia uses PP-InsPs to regulate the expression of phosphate starvation-induced (PSI) genes (Puga et al. 2014). The Mp*ITPK1* overexpression lines showed lower levels of PSI gene expression, such as Mp*SPX* and Mp*MATE1*, suggesting that PP-InsPs influence how the plant responds to Pi availability. Interestingly, in the Mp*pho1a* lines, PSI gene expression was more strongly altered in the thallus than in the rhizoids. These altered phosphate starvation responses in the mutants support the idea that PP-InsPs are crucial regulators of Pi signaling across plant species. The observed increase in various PP-InsPs in both mutants hints at a network of PP-InsPs that regulate Pi levels in Marchantia. This regulatory system likely played a key role during the transition of plants from aquatic to terrestrial environments by optimizing Pi uptake and distribution in response to limited nutrient availability in plants without true roots (Wild et al. 2016; Riemer et al. 2021).

This study demonstrates that MpITPK1 and MpPHO1a work together to regulate Pi homeostasis in Marchantia, with MpITPK1 controlling Pi-responsive signaling and MpPHO1a ensuring Pi distribution across tissues. These mechanisms in nonvascular plants parallel those in flowering plants, highlighting the evolutionary conservation of Pi management strategies (Yang et al. 2024). Additionally, MpITPK1's role in modulating PP-InsPs suggests interactions with hormone pathways, such as auxin (Laha et al. 2022) and jasmonate, which regulate growth and stress responses. Investigating these interactions may uncover new mechanisms that coordinate nutrient signaling and growth across plant species. Future research could focus on how these findings can be applied to improve nutrient use efficiency in crops, reduce fertilizer dependence, and promote growth in nutrient-poor soils.
